# Genetic Structure and Geographical Differentiation of Traditional Rice (*Oryza sativa* L.) from Northern Vietnam

**DOI:** 10.3390/plants10102094

**Published:** 2021-10-03

**Authors:** Ngoc Ha Luong, Le-Hung Linh, Kyu-Chan Shim, Cheryl Adeva, Hyun-Sook Lee, Sang-Nag Ahn

**Affiliations:** 1 Department of Agronomy, Chungnam National University, Daejeon 34134, Korea; luongngocha.biotech@gmail.com (N.H.L.); zktnrl@naver.com (K.-C.S.); ccadeva_758@yahoo.com (C.A.); leehs0107@gmail.com (H.-S.L.); 2 Agricultural Genetics Institute, North Tu Liem District, Hanoi 100000, Vietnam; lehunglinhbio@gmail.com

**Keywords:** rice, genetic diversity, population structure, genetic differentiation, marker-trait association, northern Vietnam

## Abstract

Northern Vietnam is one of the most important centers of genetic diversity for cultivated rice. Over thousands of years of cultivation, natural and artificial selection has preserved many traditional rice landraces in northern Vietnam due to its geographic situation, climatic conditions, and many ethnic groups. These local landraces serve as a rich source of genetic variation—an important resource for future crop improvement. In this study, we determined the genetic diversity and population structure of 79 rice landraces collected from northern Vietnam and 19 rice accessions collected from different countries. In total, 98 rice accessions could be differentiated into *japonica* and *indica* with moderate genetic diversity and a polymorphism information content of 0.382. Moreover, we found that genetic differentiation was related to geographical regions with an overall PhiPT (analog of fixation index F_ST_) value of 0.130. We also detected subspecies-specific markers to classify rice (*Oryza sativa* L.) into *indica* and *japonica*. Additionally, we detected five marker-trait associations and rare alleles that can be applied in future breeding programs. Our results suggest that rice landraces in northern Vietnam have a dynamic genetic system that can create different levels of genetic differentiation among regions, but also maintain a balanced genetic diversity between regions.

## 1. Introduction

Despite the controversy over its origins, rice (*Oryza sativa* L.), which is one of the most important components of the human diet, is a staple food for almost half of the world’s population [1]. Currently, rice production faces big challenges, including sustaining a growing population, conserving biodiversity, and adapting to climate change. These challenges must be tackled together for progress to be made in rice production.

How rice is bred is more relevant now than ever. Cultivated rice should produce higher yields, and genetic resources should be both efficient and resistant to biotic and abiotic stresses, such as unpredictable climate change. Among genetic resources for breeding, rice landraces have a greater genetic diversity than elite cultivars and represent an intermediate stage in domestication between wild rice and elite cultivars [2]. Rice landraces are not only more convenient to use in rice breeding than wild rice, but also maintain almost all potential sources of beneficial alleles in rice germplasm. Indeed, breeders can increase the yield of released varieties through gene pools by combining high-yielding genes/QTLs from various genetic resources of local landraces.

It is necessary to characterize and quantify genetic variability within and among populations to establish efficient strategies in breeding programs. However, thousands of valuable allelic variations of important traits remain undiscovered in nearly all crop plants. Additionally, the long-term natural selection and directional selection has led to the genetic diversity of cultivated rice, narrowing up to 80% of the wild ancestor [2]. Therefore, conserving landrace genotypes, uncovering unknown alleles, and mapping essential agronomic traits (mainly complex traits) of landraces are essential to a future full of higher yielding, better quality, and better-adapted varieties of rice. Population structure analysis provides a better understanding of genetic diversity and further information about genetic differentiation between subgroups [3,4].

Additionally, it is crucial to determine the population structure before carrying out an association analysis to reduce both type I (false-positive) and type II (false-negative) errors [5]. These errors might arise due to unequal allele frequency distributions between subgroups, which cause spurious associations between molecular markers and traits of interest [6,7,8].

Northern Vietnam is one of the most important centers of genetic diversity for cultivated rice in Vietnam and throughout the world. The “homelands” of cultivated rice stretches along the foothills of the Himalayas and the Gangetic belt up to southern China and northern Vietnam [9]. Northern Vietnam plays a significant role in these regions due to the vast genetic diversity resulting from its geographic situation and climate (varying from tropical conditions in the Red River Delta lowlands to subtropical climate conditions in the northeast highlands), the presence of many ethnic groups, and over 4000 years of rice cultivation experience [10]. Despite the abundant genetic resources, a few reports have been published relating to Vietnamese rice landraces with traits of interest, such as flowering time [11], drought tolerance [12], and salinity tolerance [13]. Recently, the resequencing of 672 native rice accessions was performed to explore genetic diversity and trait associations using the genome-wide association study (GWAS) approach [14]. The results of these studies generally recognized that Vietnamese rice germplasm had a high level of genetic diversity, and that their population structure might correlate with geographic regions. The extent of genetic differentiation between regions and the possible factors affecting the shape and population genetics of rice landraces in Vietnam remain to be determined. To ensure sufficient genetic differences in our sample, we collected rice from regions with barriers to gene flow, highly fluctuating environmental conditions, and high genetic diversity and population structure [4,15,16].

In this study, we used a new collection containing 79 rice landraces from northern Vietnam that satisfied the above conditions, 10 of which were used in a previous study by Higgins et al. (2021) [14]. Our objectives were to systematically analyze the genetic variations and population structure of this collection, select an effective marker for classifying rice (*Oryza sativa* L.) into *indica* or *japonica* subspecies, and identify the markers associated with various agro-morphological traits and emphasize the allele specificity, which are often ignored in GWAS studies. In addition, emphasis was given to provide signatures and infer explanations about the role of geographical isolation and environmental heterogeneity in genetic differentiation among regions in landraces from northern Vietnam.

## 2. Results

### 2.1. Assessment of Phenotype

**Figure 1:**
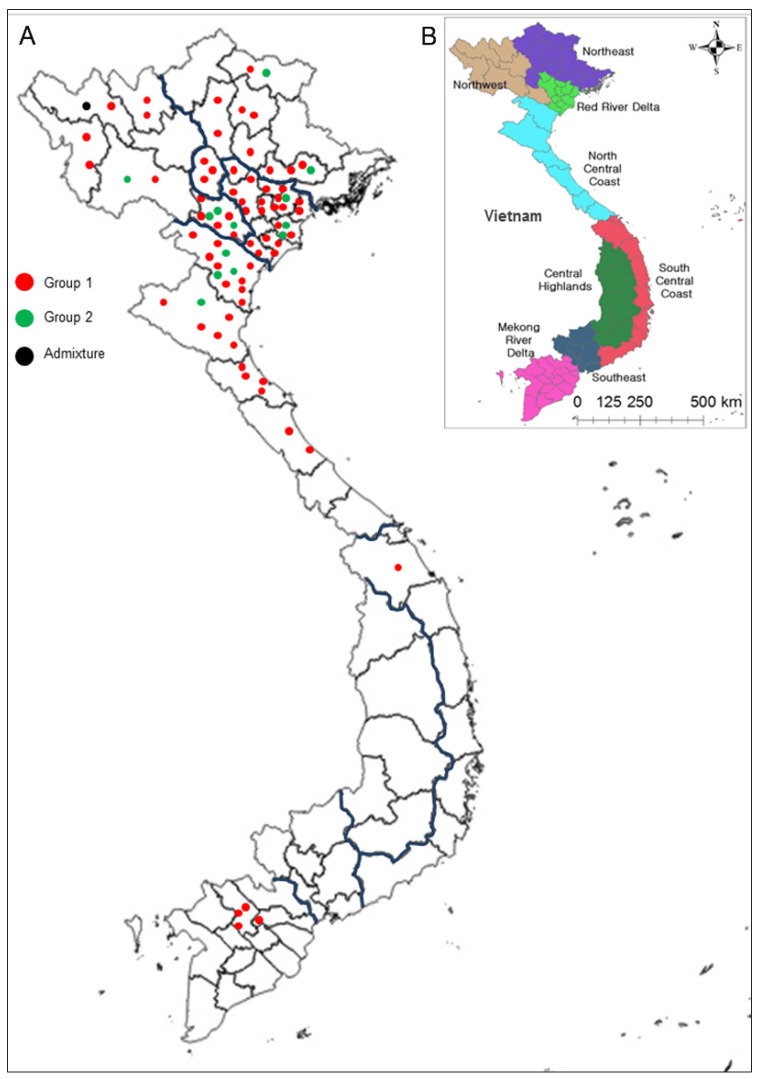
Geographic distribution of Vietnamese rice accessions in this study. (**A**) The 79 rice landraces collected from northern Vietnam belonging to AGI collection and 4 rice accessions collected from Mekong River Delta belonging to check group. (**B**) The eight main regions of Vietnam based on geographic and climatic conditions. Detailed information of the materials is provided in Table S1.

A set of 79 rice landraces collected from five ecological zones (hereafter called northern Vietnam) including the northwest (17 accessions), northeast (14), Red River Delta (26), north-central coast (21), and south-central coast (1), was used in this study (Figure 1, Table S1). This set is hereafter referred to as the “AGI collection”.

**Figure 2:**
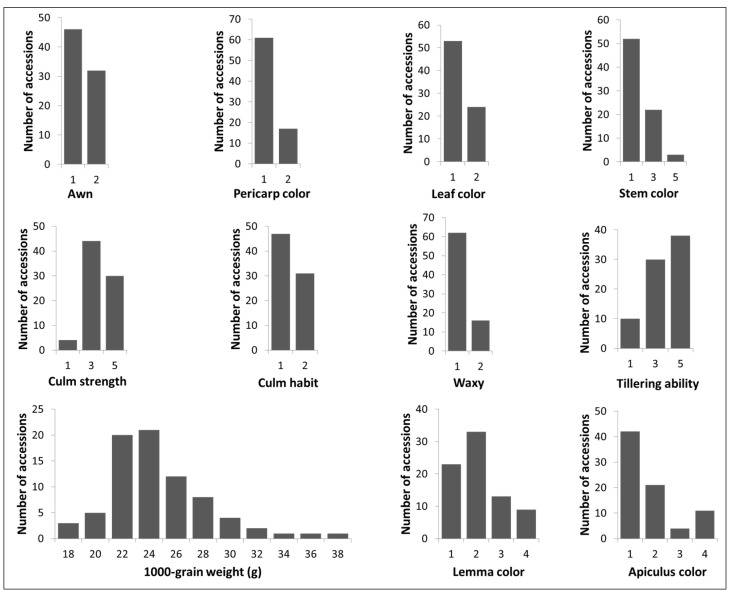
Phenotypic variation of the 11 agro-morphological traits in Vietnamese rice landraces.

A total of 11 agro-morphological traits were evaluated under field conditions in two consecutive years, 2019 and 2020. A scoring system was used for the traits including stem color and apiculus color (Table S2). The frequency distributions of 11 traits of 79 rice landraces are presented in Figure 2. Overall, 62 (78%) landraces had non-glutinous endosperm and 46 (58%) landraces were awnless. The wider ranges of variation of apiculus color and lemma color were observed. The 1000-grain weight of rice ranged from 18 to 38 g in the AGI collection. Therefore, the phenotypic variations reflected a significant amount of genetic diversity.

An image with various seed shapes and pericarp color in landraces representing for AGI collection is shown in Figure S1. It was interesting to note that AGI-25 had a long sterile lemma, a small leafy lateral organ in rice. This phenotype is possibly due to a mutation of gene(s) controlling the development of sterile lemma.

Pearson’s correlation coefficient analysis revealed relationships among the traits (Table S3). Among these, a strong correlation was observed between stem color and leaf color (r = 0.871 **), suggesting that these traits are controlled by the same anthocyanin pigmentation gene.

### 2.2. Marker Polymorphisms

To maintain the accuracy and consistency of the genetic analysis and make it comparable with previous studies, AGI collection and a ‘check group’ (including 4 accessions from Mekong River Delta and 15 accessions from other countries) were used together (Table S1). For marker selection, initially, 32 SSR markers were tested for polymorphism. Among them, only 25 SSR markers giving clear and consistent polymorphic banding patterns were used. To eliminate errors caused by genotyping, the remaining seven markers, producing ambiguous bands, were removed. In addition, 10 polymorphic InDels and 10 polymorphic Kompetitive Allele-Specific PCR (KASP) markers were added to the diversity analysis (Table S4). The genetic diversity of 98 rice accessions was evaluated using a total of 45 DNA markers. The gel pictures for banding of the 24 representative rice accessions with the markers RM70, RM287, RM201, RM475, and RM525 are shown in Figure S2. The number of alleles per locus, major allele frequency, genetic diversity, and polymorphism information content (PIC) detected among the 98 accessions are presented in Table S5. A total of 150 alleles were produced. The major allele frequency for all markers ranged from 0.219 to 0.939, with an average of 0.681. The genetic diversity of the markers varied from 0.115 (KJ06_071) to 0.880 (RM151), with an average of 0.432. The average polymorphism information content (PIC) was 0.382 with 0.108 (KJ06_071 and KJ12_055) as the lowest and 0.868 (RM151) as the highest PIC. The low heterozygosity value (0.01) in rice landraces indicated high levels of inbreeding.

Among 150 alleles, 110 alleles were amplified by SSR markers with the number of alleles ranging from 2 to 13 (an average of 4.36). The maximum number of alleles was 13 for RM151. The average PIC and GD values using SSR markers were 0.494 and 0.546, respectively. For InDel and KASP markers, the PIC values ranged from 0.108 to 0.373 with the averages of 0.277 and 0.205, respectively. Additionally, of the 150 alleles detected in 98 accessions, 24 alleles (hereafter referred to as ‘rare allele’) were identified with a low frequency (Table S6). These 24 rare alleles were detected by 14 markers, including 12 SSR markers, 1 InDel marker, and 1 KASP marker. As expected, the SSR markers in this study were more effective than the InDel and KASP markers.

### 2.3. Allele Specificity

Among the 24 rare alleles, 11 were unique in the full set of 98 accessions or unique within the AGI collection (Table S6). For example, a quick inspection revealed that a 210 bp allele (the size of PCR product for the SSR/InDel markers, same as below) amplified by the SSR marker RM424 was specific to the accession AGI-97 in the full set. Similarly, three alleles that were 260 bp, 250 bp, and 270 bp in size and amplified by the SSR marker RM421 were specific to the accessions RGW-023, RGW-043, and CNU20, respectively. The 150 bp and 330 bp alleles amplified by the SSR marker RM151 were specific to the accessions AGI-6 and AGI-98. The 320 bp allele amplified by the SSR marker RM201 was present in the accessions RGW-023, RGW-043, CNU19, and CNU20 but not in the AGI collection. In addition, 200 bp and 246 bp alleles amplified by the marker ID-J2 were shared by almost all accessions, while the 230 bp band existed only in landrace AGI-4 from the AGI collection and CNU20 (*O. rufipogon*). The allele KJ06_071-C uniquely existed in landrace AGI-4 within the AGI collection, while it was widely shared in the check group. Although visual inspection is not a comprehensive approach, the set of rare alleles in the 98 accessions could be a main factor for a high divergence score in the population structure. Moreover, these rare alleles may help the accessions survive natural selection or adapt to the demands of changing environmental conditions.

### 2.4. Population Structure Analysis

Understanding the population structure is not only important for general genetic analysis, but also for association mapping studies because it can improve the authenticity of marker-trait associations. Three methods were used to estimate the number of subgroups in the 98 accessions based on the genotype database.

**Figure 3:**
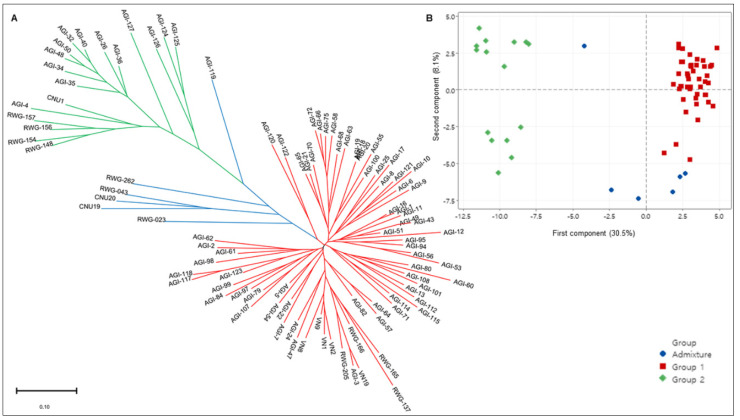
Visualization of population structure and number of subgroups within the population. (**A**) Clustering results based on Neighbor-Joining method representing Nei’s distance; (**B**) the scatter plot of first (PC1) and second (PC2) marker-derived components estimated by MINITAB software. Each point corresponds to a rice accession. Each color in (A, B) represents a subgroup.

First, cluster analysis by the Neighbor-Joining method clearly classified all 98 rice accessions into two main subgroups (defined as Group 1 and Group 2) based on their genetic background (Figure 3A). Group 1, comprising 74 accessions, was located at the lower branches of the tree, whereas Group 2, with 18 accessions, was located at the upper branch of the tree. The remaining six admixtures were in the middle of the tree. Some landraces such as AGI-18, AGI-19, and AGI-20 were clustered at the same position, although their geographical origins were different, indicating a very high level of genetic similarity. Additionally, two landraces, AGI-4 (Group 2) and AGI-119 (admixture), were separated from the other AGI collections.

Second, principal component analysis (PCA) was used to characterize the subgroups of the germplasm set. The first three principal components accounted for 45.2% of the total variability, in which the first principal components (PC1) and second principal components (PC2) explained 30.5% and 8.1%, respectively. In accordance with the cluster results, the PC1–PC2 spatial distribution map demonstrated a clear pattern of two distinct subgroups with six admixed accessions (Figure 3B).

**Figure 4:**
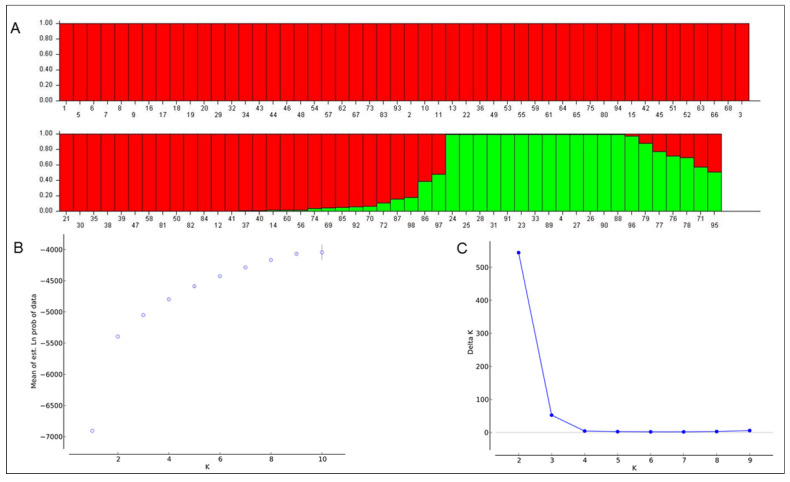
Population structure analysis of 98 rice accessions using STRUCTURE 2.3.4. (**A**) Classification of the 98 accessions into two subgroups (K = 2), (**B**) the average of log-likelihood value, and (**C**) delta K values for different numbers of populations assumed (K).

Third, the population structure of the 98 accessions was further analyzed with the software ‘STRUCTURE’ using the Bayesian clustering method. The log likelihood revealed by the structure showed an optimum value of 2 (K = 2). Similarly, the maximum of the ad hoc measure ΔK was found to be K = 2, supporting the results of the cluster analysis and PCA. Based on the membership fractions (Q value), accessions with a probability of <70% were categorized as admixtures. Most accessions were clustered into two main subgroups. The number of accessions in each group was consistent with those of the above two methods (Figure 4). The population structure matrix (Q) was generated after integrating the five replicate runs for the marker-trait association analyses (Table S1). Hence, the population structure analysis by the three classification methods revealed a high level of similarity in clustering the genotypes, which indicates that these three methods are feasible and effective.

### 2.5. Distinguishing Indica/Japonica Subspecies

To objectively categorize the AGI collections into *indica*/*japonica* subspecies, we first analyzed four subspecies-specific markers using the 98 accessions. The raw data are presented in Table S1. Based on the combined results of these markers, 61 were classified as *indica*, 16 as *japonica* subtype, and 20 as “recombinant type” The remaining landrace (AGI-119) was classified as an unknown type because it showed heterozygous bands for all four markers. This landrace might have been derived from the outcrossing between *indica* and *japonica* accessions. Moreover, AGI-119, an aromatic rice, is an intermediate type between *indica* and *japonica*. Since there is limited evidence, further studies are required to support this hypothesis. Overall, due to the high number of “recombinant type” accessions detected by the combination of four markers, further analysis, such as a comparison with population structure data, is needed to clearly distinguish these accessions. In this study, 92 accessions (except 6 accessions from the admixture group) were clearly classified into the two subgroups, which might be categorized as *indica* or *japonica*. Compared to the results of the four common markers distinguishing *indica/japonica* subspecies, we found that the result of population structure analysis in 92 accessions from Group 1 and Group 2 was the same as the result with *Pgi1*-INDEL (100%), whereas it was 84.8% for the markers ORF100 and ORF29, and 96.7% for the marker *Cat1*-INDEL, indicating that the marker *Pgi1*-INDEL is the most effective marker for classifying landrace accessions into *indica*/*japonica*.

### 2.6. Genetic Differentiation between Subgroups/Regions

Two subgroups were analyzed to characterize the genetic differentiation between and within the subgroups using the analysis of molecular variance (AMOVA). The estimated pairwise PhiPT value between Group 1 and Group 2 was 0.167, showing a high genetic differentiation with the PhiPT values significant at 0.001. However, in the total genetic variance between populations, only 17% was attributed to the subgroups and the remaining 83% was explained by individual differences within subgroups (Table 1). It indicates that a large part of the genetic background is shared by the studied accessions. In general, the results from AMOVA and pairwise PhiPT analysis were in agreement with the observations obtained through the cluster analysis, PCA, and structure analysis, confirming the presence of two main subgroups with a statistically significant differentiation.

In addition, 83 accessions (79 landraces and 4 improved rice) from Vietnam were further divided into five groups according to geographic region: northwest (17 accessions), northeast (14), Red River Delta (26), north-central coast (21), and southern Vietnam (5) (Figure 1, Table S1). The 15 remaining accessions from the check group were gathered together as an “international group” (Table S1). There were some distinct differences in allele frequencies among the 98 rice accessions from different locations (Table S7). For example, the allele frequency of RM278-155 (the size of PCR product for each marker, same as below) varied from 8.3% (northwest, NW) to 50% (north-central coast, NCC), and the frequency of ID-Glu23-220 varied from 11.8% (NW) to 47.1% (northeast). Additionally, some specific alleles could be detected only in Vietnamese landraces or only in international rice accessions. RM21-165 was distributed only in all five regions of Vietnam, but not in international rice accessions. In contrast, alleles RM70-220 and RM201-320 could only be detected in the international rice accessions. Notably, some region-specific alleles among Vietnamese landraces in the four main regions were observed. Alleles RM21-140, RM287-95, and KJ06_071-C were detected only in the Red River Delta region, with the allele ID-J2-200 only in the northwest. This result is consistent with the overall trend in the Neighbor-Joining tree (Figure 3A). Most of the accessions from the international group were slightly favored to be group together than with landraces from northern Vietnam. 

To test the above results, the AMOVA analysis was also performed among six geographical regions to check whether there was significant genetic differentiation among regions. The results of AMOVA statistics depicting the degree of differentiation among accessions from different regions are shown in Table 2. Within the region, variation accounted for 87% of the total variation, indicating a lager contribution of variation within the region. Additionally, a moderate degree of genetic differentiation was observed among the six regions, with an overall PhiPT value of 0.130. Pairwise PhiPT values between regions varied from 0.040 to 0.220. Very high PhiPT values were observed between the Red River Delta region with both the international group (0.220) and southern Vietnam (0.200). A low PhiPT value (0.078) was observed in accessions from the international group and southern Vietnam. Among Vietnam accessions, the PhiPT value (0.097) between southern Vietnam and north-central coast was lower than that between southern Vietnam and other regions such as the Red River Delta (PhiPT = 0.200), northwest (PhiPT = 0.125), and northeast (PhiPT = 0.155). Among landraces from AGI collection, the Red River Delta showed moderate (PhiPT = 0.067) to high (PhiPT = 0.194) genetic differentiation with the northwest and northeast regions, respectively. Likewise, the north-central coast region showed moderate (PhiPT = 0.052) to relatively high (PhiPT = 0.116) genetic differentiation with the northwest and northeast regions, respectively. Notably, a very high PhiPT value (0.188) was observed between the northwest and northeast regions, while the lowest PhiPT value (0.04) was observed between the Red River Delta and the north-central coast. We noted that the different climatic and geographical conditions between regions in this study could have affected population genetics. The northwest and northeast regions were separated by the Hoang Lien Son Mountains, leading to geographical isolation, which could be a factor affecting the high genetic differentiation between these regions. Additionally, the presence of many ethnic minorities that developed their own rice culture and reused seeds from the previous harvest might be another factor.

### 2.7. Genetic Diversity within Subgroups and Geographic Distribution of Genetic Diversity

Genetic diversity of the two subgroups and six geographic regions were compared using two parameters: the average value of gene diversity (GD) and the average value of PIC (Table 3).

In the full set, Group 1, which was composed of 74 *indica* accessions, displayed slightly lower GD and PIC values than Group 2 with 18 *japonica* accessions. In contrast, in AGI collection, Group 1 (65 *indica* landraces) displayed a slightly higher genetic background than Group 2 (13 *japonica* landraces). When the GD of the six groups by geographic region was compared, the international group showed the highest diversity, while the southern Vietnam accessions displayed the lowest. The GD of the four groups from AGI collection, in general, was slightly different: northwest > north-central coast > northeast > Red River Delta. We tested the statistical significance of the difference in GD and PIC between the two subgroups as well as between the six regions, and the results indicated that the difference in GD and PIC values between the two subgroups or between each pair of regions were not statistically significant, except for the GD and PIC values of the southern Vietnam group (consisting of four improved rice and one landrace), which were significantly (*p* < 0.001) lower than those of the other five regions. We found that, although high genetic differentiation was found among the geographic regions in northern Vietnam, especially between the Red River Delta and northeast, the genetic diversity among the four geographic regions was similar. Notably, GD and PIC values widely agreed. These values reflect the absolute number of alleles segregating within a population/subpopulation, considering their frequencies. In contrast, genetic differentiation universally compares the differences in genetic variation between two or more parts of a population, which depends on the relative frequencies of the allelic types and genetic distances [17]. Our results suggest that rice landraces in northern Vietnam have a dynamic genetic system that can create different levels of genetic differentiation among regions while maintaining balanced genetic diversity between regions. 

### 2.8. Contribution of Population Structure to Phenotypic Variation

The t-test was employed to determine whether significant differences between the two subgroups of AGI collection existed for 11 phenotypic traits (Figure S3). The results revealed highly significant phenotypic differences (*p* < 0.05) between Group 1 and Group 2 in most morphological traits, including stem color and culm strength. The image showing various shapes of seeds and grain pericarps in rice landraces representing Groups 1 and 2 is shown in Figure S1. In general, the seed shape of landraces in Group 1 showed typical characteristics of *indica* rice, excluding AGI-112, showing typical *japonica* characteristics. The seed shape of most landraces in Group 2 displayed typical *japonica* characteristics, excluding AGI-124, AGI-125, AGI-126, and AGI-127, which showed larger grains. We noted that these four *japonica* landraces carried RM242-190, RM242-195, and RM277-130 alleles, which were popular in *indica* landraces, whereas the remaining *japonica* accessions carried specific alleles at these loci, which were rare in *indica* accessions (Table S8). The results indicated that some gene/QTLs are possibly responsible for the grain size in these landraces.

### 2.9. Association Analysis within AGI Collection

Given that population structure is the main factor affecting marker-trait association, the association between markers and the phenotypic data of the 11 traits in AGI collection was examined using three different models: naive generalized linear model (GLM), GLM with Q, and mixed linear model (MLM) with Q + K. Further, these three models were compared for best fit using a quantile–quantile (Q-Q) plot; the MLM with Q + K model was the best model fitted in reducing false-positive association results (Figure S4). All of the marker-trait associations detected in MLM were also detected in the GLM at *p* < 0.01 (Table S9). Therefore, the results under the optimal model MLM with Q + K (hereafter referred to as MLM) were used in this study.

**Figure 5:**
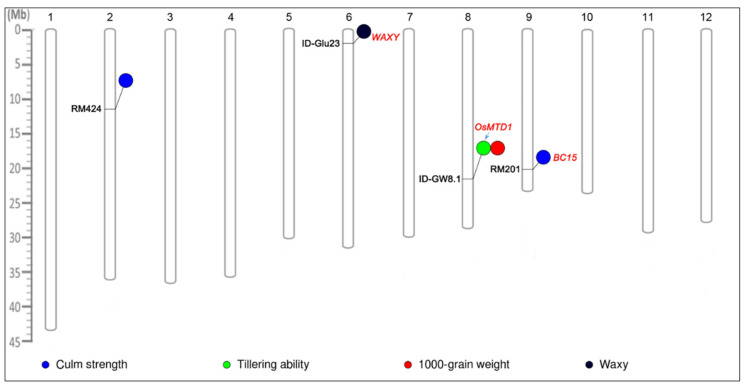
Physical locations of the significant marker-trait association detected by MLM (Q + K) based on the tool PhenoGram (http://visualization.ritchielab.psu.edu/ (accessed on 3 July 2021)). Each chromosome was manually rearranged from bottom to top based on a ruler.

A total of five significant marker-trait associations were detected using MLM in the AGI collection (*p* < 0.01) (Figure 5). Of these, two marker-trait associations were detected for culm strength, one for tillering ability, one for 1000-grain weight, and one for the waxy phenotype. Marker ID-Glu23 on chromosome 6 showed the strongest association with the waxy phenotype, with a phenotypic variance (PV) of 34.3%. In addition, the SSR marker RM201 was associated with culm strength with PV of 13.8%. MLM also detected the association of ID-GW8.1 with tillering ability and 1000-grain weight.

### 2.10. Performance of Traits Relevant to Different Alleles of Significant Loci

Tukey’s test was used to compare the phenotypic means of genotypic classes at the two selected loci (Table S10). The marker ID-GW8.1 was associated with tillering ability and 1000-grain weight. At this locus, accessions carrying a 240 bp allele had significantly (*p* < 0.001) higher tillering ability but lower grain weight than those with the 210 bp allele. At SSR marker RM424, 7 accessions with a 160 bp and 13 accessions with a 130 bp had a significantly stronger culm than those accessions carrying 170 bp or 180 bp alleles. Additionally, we noted that 13 accessions of 130 bp were *japonica*, while the remaining accessions carrying 160 bp, 170 bp, or 180 bp were *indica* (Table S8).

## 3. Discussion

### 3.1. Genetic Diversity

The study of genetic diversity can lead to the utilization, conservation, and management of valuable resources like rice germplasm; it can be applied in future breeding programs and molecular approaches. A wide range of phenotypic distribution was observed among 11 traits in the AGI collection, reflecting a significant amount of genetic diversity (Figure 2). However, the diversity of the accessions in this study (with mean values of 0.432 and 0.382 for genetic diversity and PIC, respectively) was relatively lower than that reported in previous studies with PIC values of 0.468 [3] and 0.725 [4]. This was expected because the bi-allelic nature of the SNPs detected by KASP markers or the deletion/insertion detected by InDel markers restricted the maximum PIC values to 0.5 (when the two alleles have identical frequencies), reduced the mean of genetic diversity, and reduced the PIC value (Table S5). Additionally, this might be due to the narrow geographical range (mostly from northern Vietnam) of rice accessions used in this study as compared to other research studies (mostly collected across the country, but including some from across the world). Frequently, the degree of genetic diversity has been highly correlated with the geographical origin of the accessions, indicating that the greater the number of accessions, the greater the genetic diversity [18,19]. 

### 3.2. Population Structure and Factors Affecting Genetic Differentiation

Rice (*Oryza sativa* L.) has two main subspecies, *indica* and *japonica*, which are distantly related in terms of their genetic relationships. Therefore, it was not surprising that the *indica* and *japonica* subgroups were identified in this study. Additionally, the population structure analysis revealed a high level of consistency and accordance in clustering the genotypes, indicating the reliability and effectiveness of our genotyping approach and providing a reliable method of determining population structure to facilitate subsequent marker-trait association studies. Moreover, these results are like those of previous studies, in which the population structure analysis revealed that the germplasm lines were grouped into two distinct subgroups [3]. Coincidentally, the inferred ancestry values of IR64 (0.995–0.005) were the same as those detected in our study (Table S1). This confirms the reliability and accuracy of our genotypic data and population structure results.

Based on Wright’s (1978) qualitative guidelines for the interpretation of F_ST_ [20], a range from 0 to 0.05 indicates little genetic differentiation, whereas the ranges 0.05–0.15, 0.15–0.25, and >0.25 indicate moderate, great, and very great genetic differentiation, respectively. In this study, the PhiPT value (an analog of fixation index F_ST_) between Groups 1 and 2 was 0.167, indicating high differentiation between the two subgroups. Additionally, to complement the above results of the population structure, the AMOVA analysis was also performed in six geographical regions to check whether the genetic differentiation among accessions was explained by their geographical regions. Although the PhiPT value was lower than that between the two subgroups, moderate genetic differentiation was observed among the six regions with an overall PhiPT value of 0.130 (Table 2).

Genetic differentiation among the six regions implies the correlation with their geographical locations. For this reason, we must ask what factors influence genetic differentiation among these regions. Both the international and southern Vietnam groups showed higher genetic differentiation than the other groups from northern Vietnam. However, little genetic differentiation (PhiPT = 0.078) was observed between the international and southern Vietnam groups, possibly because several improved rice varieties were shared by both groups. Furthermore, some accessions of the southern Vietnam group were derived from crosses with rice accessions from the International Rice Research Institute (IRRI), such as IR 63356-6B, as the parent of VN2 in this group. Additionally, IR64 and several rice varieties from IRRI were widely used in breeding programs in southern Vietnam after the Green Revolution. Among the three groups—the Red River Delta, north-central coast, and southern Vietnam—the lowest PhiPT value (0.040) was observed between the Red River Delta and the north-central coast; a higher PhiPT value (0.097) was observed between the north-central coast and southern Vietnam, and the highest PhiPT value (0.200) was observed between the Red River Delta and southern Vietnam. Taken together, the genetic differentiation among groups had a positive correlation with their geographical distances, wherein the likelihood of gene flow was inversely related to the physical distance between regions. Notably, this was seen between the northern Vietnam groups, where the Red River Delta and north-central coast showed moderate to great (PhiPT values ranged from 0.052 to 0.194) genetic differentiation in the northwest and northeast. The Red River Delta and north-central coast belong to the lowland eco-region with a tropical monsoon climate, while the northwest and northeast regions belong to highland eco-regions with subtropical climate, especially the northeast regions. Therefore, the differentiation between accessions from highlands and lowlands could be explained by local selection pressures. Additionally, a very high PhiPT value (0.188) was observed between the northwest and northeast regions, while the lowest PhiPT value (0.04) was observed between the Red River Delta and the north-central coast. The reason for this differentiation was not only due to environmental conditions, but also to gene flow from seed exchange among farmers. The Red River Delta and north-central coast are mainly plains, resulting in a more convenient seed exchange network among farmers in these areas. This may facilitate gene flow. In contrast, the northwest and northeast regions are separated by the Hoang Lien Son mountains (end of the Himalayas), which somewhat limits the connectivity between the two regions. The significant genetic differentiation between northwest and northeast landraces was a sign of a reduction in gene flow between these regions. Most likely, the Hoang Lien Son mountains (including Mount Fansipan peak, which at 3143 m is the highest point in the Indochina region) act as a barrier to gene flow. Another possible explanation for the differentiation between the northwest and northeast regions might be the presence of many ethnic minorities that developed their own rice culture and have solely reused seeds from the previous harvest. The genetic differentiation among the six regions was correlated with their geographical locations due to local selection pressures and seed dispersal by humans who brought seeds for cultivation and/or who exchanged seeds with other regions. Similar findings were reported in a previous study in which cultivation practices such as seed exchange between close villages and selection by farmers played a predominant role in reducing the genetic differentiation in the rice landrace Bue Chomee in Thailand [15]. Recently, another study demonstrated that geographic isolation and environmental heterogeneity were important in the population structure of rice landraces in Yunnan Province, China [4]. Both previous studies attested our evidence in the case of Vietnamese rice landraces.

Interestingly, we found that genetic differentiation between geographic regions was not correlated with genetic diversity within geographic regions. Although a high level of genetic differentiation was found among the geographic regions, especially between the northeast and the other three regions in northern Vietnam, similar genetic diversity was observed within each geographic region. A possible explanation for this phenomenon could be that the genetic differentiation between regions was mainly affected by gene flow and limited by barriers, such as high mountains or distance between regions, while genetic diversity within regions could be mainly driven by other factors, such as adaptation to local environmental heterogeneity within each region (such as between fields or provinces) and selection pressure. The results of our study suggest that cultivation relies on the natural environment not only to create different levels of genetic differentiation among regions, but also to maintain a balanced genetic diversity with nature. This is the importance of cultural practices in maintaining the diversity and local environmental adaptations of crop germplasm [15].

### 3.3. Distinguishing Indica and Japonica Subspecies

Quick and accurate classification of rice germplasms into *indica* or *japonica* can provide essential information for parental selection strategies in rice breeding programs. Previously, most studies strongly agreed that the population structure of rice accessions (*Oryza sativa* L.) could be classified into two main subgroups corresponding to *indica* or *japonica* [11,12,14]. However, a previous study detected 10 *indica* accessions classified together with the subgroup (SG2) as dominated by *japonica* accessions [3]. To explain this phenomenon, morphological and physiological characteristics including apiculus hair length, leaf color, glume color at heading stage, grain length-width ratio, and phenol reaction were also employed to classify *indica* and *japonica* [21,22,23]. At the same time, population structure can be detected based on DNA markers. Comparatively, DNA markers are more reliable, as they provide systematic results.

Four specific markers (one chloroplast marker ORF100 and three nuclear markers, *Pgi1*-INDEL, *Cat1*-INDEL, and *Acp1*-INDEL) were used to classify the subspecies in a previous study [24]. A total of 35 accessions were classified as *japonica*, 28 as *indica*, and some of the accessions as “recombinant type” based on the combined genotypes of four InDel markers. However, two distinct groups corresponding to *indica* and *japonica* groups were obtained using PCA based on 12 SSR markers. Another study detected more “recombinant type” accessions based on the combined genotypes of the same set of markers [25]. The same phenomenon was observed in the present study (Table S1). Therefore, it is necessary to select a specific marker to classify rice into *indica* or *japonica* effectively.

In this study, 92 accessions (except 6 from the admixture group) were classified into two subgroups: *indica* or *japonica*. In comparison to the results for each of the four common markers, population structure analysis showed the following results: 100% consistent with *Pgi1*-INDEL, 84.8% with ORF100 and ORF29, and 96.7% with *Cat1*-INDEL, indicating that *Pgi1*-INDEL was the most effective marker for classifying rice accessions into *indica*/*japonica*. To the best of our knowledge, this is the first study to compare the specificity of these markers with population structure analysis. Since a small number of rice accessions were used in this study, it is necessary to employ more samples to support our findings.

### 3.4. Marker-Trait Association Analysis

A small number of markers were not sufficient for QTL mapping or for identifying novel genes effectively within the framework of this study. Thus, we investigated marker-trait associations to provide and gather initial information that can be applied in future breeding programs.

Given that population structure and relatedness are the main factors that can provide authenticity of marker-trait association, we conducted an association analysis between DNA markers and 11 agro-morphological traits using the GLM (Q) and MLM (Q + K) models. The comparison between these two models showed that MLM, which accounted for both population structure and kinship, decreased the total number of significant associations. All of the marker-trait associations detected in MLM were also detected in the GLM at *p* < 0.01 (Table S9). As was pointed out in the paper by Yu et al. [5], the MLM model was better in correcting false-positive associations than GLM. This is consistent with our results (Figure S4). Therefore, the results under the optimal MLM model were used in this study.

A total of five significant marker-trait associations were detected using MLM within the AGI collection (*p* < 0.01). Among these, we noticed that the identified genetic locus for starch biosynthesis genes *Wx* (*Os06g0133000*) [26] was colocalized with the locus *ID-glu23*. We also found a locus *ID-GW8.1* for tillering ability in the nearby region of *OsMTD1* (*Os08g0441300*), a gene regulating tillering ability and plant architecture in rice [27]. The significant association locus RM201 for culm strength was found close to the identified gene *OsBC15* (*Os09g0494200*), a brittle culm gene [28]. These known genes detected in previous studies could also provide evidence for our marker-trait association identification. The remaining markers, such as RM424 for culm strength and ID-GW8.1 for 1000-grain weight, were detected on chromosomes 2 and 8, respectively. However, it is not sufficient to identify putative candidate genes; hence, additional research studies, such as QTL mapping, cloning, and functional validation, are required in the future.

### 3.5. Rare Allele

Favorable alleles of some major genes associated with traits of agronomic importance tend to be ubiquitous in many modern rice varieties; hence, these alleles are not highly effective in breeding programs. Exploring rare germplasm and identifying specific alleles are required for adapting to climate change and developing novel breeding tools [29].

Of the 150 alleles detected in 98 accessions, 24 alleles were identified with a low frequency (<0.05) by 14 markers, including 12 SSR markers, 1 InDel marker, and 1 KASP marker (Table S6). As expected, the SSR markers in this study showed higher efficiency than the InDel and KASP markers. Among the 14 markers, the 200 bp and 246 bp alleles by ID-J2 were shared by almost all accessions, while the 230 bp allele existed as a rare allele only in landrace AGI-4 and CNU20 (*O. rufipogon*). Similarly, the allele KJ06_071-C uniquely existed in landrace AGI-4, while it was widely shared in the check group. In addition, the landrace AGI-4 was located separately from the others in AGI collection on the NJ tree (Figure 3A) but was located near CNU-1 (Nipponbare), which was used as a reference genome [30]. Interestingly, in terms of phenotype, the landrace AGI-4 showed some characteristics similar to wild rice, such as purple pericarp, purple apiculus, and long and purple awning (Figure S1), which may be controlled by rare alleles, such as the 230 bp allele detected by markers ID-J2 or the KJ06_071-C allele. Unfortunately, rare accessions with unique alleles may be neglected due to the lack of interest in plant breeders, which may harbor genetic information worth conserving. It is also important to mention that allele specificity can be valuable in mining rare alleles associated with specific adaptations. Overall, discovering highly informative rare alleles will provide more powerful genetic tools to detect the loci-trait association signal. Based on these results, we suggest that further studies should develop a population derived from a cross between landrace AGI-4 and Nipponbare to map genes associated with specific traits.

Additionally, based on the observed seed shape, there was one exception (AGI-112), which shows the typical characteristics of *japonica* (Figure S2). However, some agro-morphological traits of AGI-112 showed the typical characteristics of Group 1, such as light green stems and leaves and strong tillering ability. Moreover, AGI-112 is an *indica* based on the results of STRUCTURE analysis (inferred ancestry of 0.997) and marker *Pgi1*-INDEL (Table S1). One possible reason might be mutated alleles at the gene(s) controlling seed shape. Further studies, including gene mapping and transgenic approaches, are needed to characterize the genetic mechanism of grain shape in AGI-112. Additionally, four *japonica* landraces, AGI-124, AGI-125, AGI-126, and AGI-127, had significantly larger grains than the other *japonica* accessions. Genotype analysis showed that these four *japonica* landraces carried different alleles at RM242 and RM277 than other remaining *japonica* accessions (Table S8). We also found that the identified genes for grain size *OsFH15* (*Os09g0517600*) [31] and QTL *gw9.1/gw9* for both grain shape and grain weight [32,33] on chromosome 9 were close to the marker RM242. Similarly, another QTL for grain length *qGL12.1* [33] on chromosome 12 was detected near RM277. Therefore, these gene/QTLs are possibly responsible for the large grain size in the four *japonica* landraces.

## 4. Materials and Methods

### 4.1. Plant Materials

A set of 79 rice landraces collected from five ecological zones (called northern Vietnam) including the northwest (17 accessions), northeast (14), Red River Delta (26), north-central coast (21), and south-central coast (1), was used in this study (Figure 1, Table S1). These landraces were held in the Genebank of the Molecular Biology Division of the Agricultural Genetics Institute (AGI; Hanoi, Vietnam). This set is referred to as the “AGI collection”. Another set consisting of 19 accessions was chosen (Table S1). This set is referred to as the “check group”.

### 4.2. Phenotypic Screening and Statistical Analysis

Phenotypes of AGI collection for agro-morphological traits were screened under field conditions at the experimental farm of AGI (Van Giang, Hung Yen province, Vietnam) in two consecutive years, 2019 and 2020. The field experiment was laid out in a completely randomized block design with two replications. Ten agro-morphological traits controlled by major genes and one quantitative trait, 1000-grain weight, were evaluated according to the Standard Evaluation System for Rice (SES) with slight modifications (Available online: http://www.knowledgebank.irri.org/images/docs/rice-standard-evaluation-system.pdf (accessed on 11 May 2019)). Ten plants of each accession were used to collect the phenotypes. For the 1000-grain weight, grains harvested from 10 plants were mixed and 100 randomly selected grains were measured in grams with three replicates, and then the average was multiplied by 10. A scoring system was used for the following qualitative traits: waxy, awning, culm habit, leaf color, stem color, culm strength, tillering ability, lemma color, apiculus color, and pericarp color (Table S2). The frequency distributions of the 11 traits were constructed using GenAlEx, version 6.503 [34].

Pearson’s correlation coefficient was calculated, and the correlation thresholds were considered significant at *p* < 0.01 using MINITAB 16.2.4.

### 4.3. SSR and InDel Markers Analysis

A total of 98 rice accessions were genotyped using 35 DNA markers: 25 SSR and 10 InDel markers. Total genomic DNA was isolated from young leaves of each accession using the cetyltrimethylammonium bromide (CTAB) method with minor modifications [35]. DNA quantification was performed using a spectrophotometer at wavelengths of 260 and 280 nm (Nanodrop ND-1000, Thermo Fisher Scientific, Waltham, MA, USA). The final DNA concentration of each sample was diluted to a working concentration of 20 ng/µL and used for PCR amplification. The PCR reaction mixture was prepared as previously described [36]. The PCR products were run on a 3% high-resolution MetaPhor agarose gel (Lonzan, Rockland, ME, USA) and visualized with a UV transilluminator. To maintain the accuracy and consistency of band size measurements, the alleles of four samples (Milyang 23, Nipponbare, *O. rufipogon,* and AGI-1 from AGI collection) and a 100 bp DNA ladder (Invitrogen, USA) were used to estimate the allele molecular weight in every 24 samples. The allele score was given based on the presence of a particular size allele in each of the accession (Table S11). Information on all primers used in this study is presented in Table S4.

### 4.4. KASP (Kompetitive Allele-Specific PCR) Analysis

All 98 rice accessions were genotyped using 10 KASP markers at the Seed Industry Promotion Center, Foundation of Agri. Tech. Commercialization and Transfer, Korea. The KASP analysis was carried out based on the method described by Cheon et al. (2018) and Yang et al. (2019) [37,38].

### 4.5. Distinguishing Indica-Japonica Subspecies

To discriminate the Vietnamese rice accessions into *indica/japonica*, another set of four common markers, two chloroplast InDel markers (ORF100 and ORF29 [39,40]) and two nuclear InDel markers (*Pgi*1-INDEL and *Cat*1-INDEL, designed based on the isozyme loci coding for phosphoglucose isomerase and catalase [41], respectively) were employed.

### 4.6. Genetic Diversity and Population Structure Analysis

Genetic diversity parameters, such as number of alleles per locus, major and minor allele frequency, availability, genetic diversity, heterozygosity, and PIC, were calculated using the program ‘POWERMARKER’ software V 3.25 [3,4,42,43]. Subsequently, the genetic distance matrix was computed using the NEI coefficient [44] with the bootstrap procedure of resampling (1000). To determine the association among the accessions, an unrooted Neighbor-Joining phylogenetic tree was also drawn using POWERMARKER and viewed in MEGA 6.0, with 1000 bootstrap replicates [45].

PCA was performed to study the relationships among the accessions using genetic data from 45 DNA markers. PCA was performed using the MINITAB 16.2.4.

The population structure was deduced from a model-based approach using STRUCTURE V 2.3.4 software [3,6,46,47]. The project was set with a run length of 100,000 burns-in, followed by 100,000 Markov Chain Monte Carlo simulations. Five independent iterations of running were performed with the optimum number of subgroups (K) ranging from 1 to 10. The correct estimation of K was determined using ad hoc statistics ∆K proposed by Evanno et al. [48] in the structure harvester [49]. Accession was classified in a subgroup if it had the highest membership (inferred ancestry >70%) coming from that group; otherwise, it was classified as an admixture.

Genetic differentiation between the two subgroups was estimated by AMOVA based on the NEI distance matrix obtained from POWERMARKER software to justify the suitability of group classification using GenAlEx, version 6.503 [3,34,46,47] with 999 permutations. The level of genetic differentiation between subgroups was determined using PhiPT in AMOVA, a measure that provides important insights into the evolutionary processes that influence the structure of genetic variation within and among subgroups [50,51]. PhiPT values [*V*_AP_/(*V*_WP_ + *V*_AP_)] denote the proportion of the variance among subgroups relative to the total variance between subgroups. *V*_AP_ and *V*_WP_ are the estimates of variance among and within subgroups, respectively. The higher PhiPT values are indicative of the greater differences between subgroups. To complement the above results of population structure, the AMOVA analysis was also performed among these six geographical regions to check whether the genetic differentiation among accessions was explained by their geographic regions.

In addition, the two-sided t-test was performed on 11 agro-morphological traits to compare means between the two subgroups using MINITAB 16.2.4.

### 4.7. Marker-Trait Associations

Marker-trait association was performed based on the phenotypic data, genotypic data, and Q matrix of AGI collection using the software TASSEL 5 v.5.2.72 [46,52]. The mean value of 1000-grain weight of each landrace was calculated using 2-year data for association analysis. To obtain a reliable association, markers with more than 10% missing data and/or minor allele frequencies less than 5% were omitted. Heterozygous genotypes were also considered missing data. After filtering, 41 markers distributed across 12 rice chromosomes were retained for further analysis. To control false-positive results, the association of markers with traits was determined using a GLM (Q) to correct the population structure, and a MLM (Q + K) to correct both the population structure and relative kinship [5]. To check the effect of these two models, a naive GLM was also conducted. Markers were defined as being significantly associated with traits when the *p*-value was less than 0.01.

The physical locations of the significant markers identified by association analysis were mapped on the 12 rice chromosomes using the web-based tool PhenoGram (http://visualization.ritchielab.psu.edu/ (accessed on 3 July 2021)), and each chromosome was manually rearranged from bottom to top based on a ruler.

Tukey’s test in MINITAB was implemented to compare the performance of agronomic traits related to different alleles of significant marker-trait associations.

## 5. Conclusions

In the present study, we analyzed genetic diversity, population structure, and marker-trait associations in rice landraces from northern Vietnam. The rice landraces could be differentiated into *japonica* and *indica* groups with moderate genetic diversity. Moreover, we found that the level of genetic differentiation was related to location within northern Vietnam. Additionally, we detected a subspecies-specific marker to effectively classify rice (*Oryza sativa* L.) into *indica* or *japonica*. By analyzing marker-trait associations, we detected five marker-trait associations and rare alleles that can be applied in future breeding programs and molecular approaches. Our results suggest that rice landraces in northern Vietnam have a dynamic genetic system that can create different levels of genetic differentiation among regions while maintaining balanced genetic diversity between regions.

## Figures and Tables

**Table 1:** Summary of AMOVA and pairwise comparison using PhiPT values among and within subgroups.

Source	df ^1^	SS	MS	Est. Var.	Percent
Among subgroups	1	1.350	1.350	0.040	17%
Within subgroup	90	17.852	0.198	0.198	83%
Total	91	19.202		0.238	100%
Pairwise population PhiPT values ^2^					
	Group 1	Group 2			
Group 1	0.000	0.001			
Group 2	0.167	0.000			

^1^ df, degrees of freedom; SS, sum of squares; MS, mean sum of squares; Est. Var., estimate of variance; Percent, percentage of total variation. ^2^ PhiPT values below diagonal. Probability based on 999 permutations is shown above diagonal.

**Table 2:** Summary of AMOVA and pairwise comparison using PhiPT values among and within regions.

Source	df ^1^	SS	MS	Est. Var.	Percent	Overall PhiPT	Probability
Among regions	5	3.242	0.648	0.029	13%	0.130	0.002
Within region	92	17.774	0.193	0.193	87%		
Total	97	21.016		0.222	100%		
Pairwise population PhiPT values							
	NW ^2^	NE	RRD	NCC	S	Inter	
NW	0.000						
NE	0.188	0.000					
RRD	0.067	0.194	0.000				
NCC	0.052	0.116	0.040	0.000			
S	0.125	0.155	0.200	0.097	0.000		
Inter	0.190	0.104	0.220	0.135	0.078	0.000	

^1^ df, degrees of freedom; SS, sum of squares; MS, mean sum of squares; Est. Var., estimate of variance; Percent, percentage of total variation; Overall PhiPT, genetic differentiation among regions. Probability based on 999 permutations. ^2^ NW, northwest; NE, northeast; RRD, Red River Delta; NCC, north-central coast; S, southern; Inter, international.

**Table 3:** The average number of alleles, GD, and PIC for different subgroups/regions.

Groups	Statistics ^1^					
	MAF	SS	Na	GD	He	PIC
Full set	0.681	98	3.333	0.432	0.011	0.382
Group 1 (*indica*)	0.777	74	2.978	0.306 ^A^	0.011	0.273 ^A^
Group 2 (*japonica*)	0.758	18	2.356	0.324 ^A^	0.004	0.276 ^A^
Admixture	-	6	-	-	-	-
AGI collection	0.700	79	3.156	0.402	0.012	0.356
Group 1 (*indica*)	0.776	65	2.867	0.299 ^A^	0.012	0.268 ^A^
Group 2 (*japonica*)	0.776	13	2.178	0.294 ^A^	0.005	0.252 ^A^
Admixture	-	1	-	-	-	-
Six regions						
Northeast	0.718	14	2.533	0.376 ^A^	0.003	0.327 ^A^
Northwest	0.677	17	2.733	0.424 ^A^	0.016	0.366 ^A^
Red River Delta	0.725	26	2.867	0.367 ^A^	0.016	0.326 ^A^
North-central coast	0.698	21	2.867	0.400 ^A^	0.012	0.351 ^A^
Southern	0.880	5	1.467	0.166 ^B^	0.004	0.136 ^B^
International	0.615	15	2.800	0.473 ^A^	0.005	0.406 ^A^

^1^ MAF, major allele frequency; SS, sample size; Na, number of alleles; GD, genetic diversity; He, heterozygosity; PIC, polymorphism information content. The numbers followed by different letters in a column for each data set are significantly different at *p* < 0.05, based on Tukey’s test.

## Data Availability

All data supporting the findings of this study are available within the paper and within its supplementary data published online.

## Supplementary Materials

The following are available online at https://www.mdpi.com/article/10.3390/plants10102094/s1, Figure S1: Photograph showing various appearances of seeds and grain pericarp in rice landraces representative of Group 1 and Group 2. The gray circle with a diameter of 2.2 cm is used as a scale bar, Figure S2. Gel pictures for banding of the 24 representative rice accessions with the markers: (A) RM70, (B) RM201, (C) RM287, (D) RM475, and (E) RM525. #1—Milyang 23; #2—Nipponbare; #3—*O. rufipogon*; from #4 to #24: AGI-1 to AGI-24, Figure S3: Comparison of the means for 11 agro-morphological traits among two subgroups by t-tests, Figure S4: Quantile–quantile (Q–Q) plot of the marker-trait association by naive GLM, GLM with Q, and MLM with Q  +  K for culm habit, waxy, and stem color, Table S1: Description of 98 rice accessions used for this study and raw data obtained from STRUCTURE and four subspecies-specific markers, Table S2: Description of scoring system, Table S3: Correlation coefficients among 11 traits in Vietnamese landraces, Table S4. Characteristics of 45 DNA markers used for this study, Table S5: Genetic diversity characteristics of 45 DNA markers, Table S6: A list of accessions with rare alleles in this study, Table S7: Comparison of specific allele frequency (%) between regions, Table S8: Genotype of *indica/japonica* landraces at selected loci (RM242, RM277, and RM424), Table S9: Significant marker-trait associations in AGI collection detected by GLM (Q) and MLM (Q + K) models, Table S10: Multiple comparisons for significant marker-trait association detected by MLM (Q + K), Table S11. Allele matrix of 98 rice accessions x 45 DNA markers.
