# Supplementary material for: Genetic Structure and Geographical Differentiation of Traditional Rice (Oryza sativa L.) from Northern Vietnam
Source: Plants (Basel). 2021 Oct 3;10(10):2094. doi: 10.3390/plants10102094 (PMC8540186; doi:10.3390/plants10102094)
Supplement: Supplementary file 1 [file plants-10-02094-s001.zip › plants-1391621-supplementary/Figure S1-S4.pdf]

Supplementary Files

Genetic Structure and Geographical Differentiation of Traditional Rice (*Oryza sativa* L.) from northern Vietnam

Ngoc Ha Luong [1], Le-Hung Linh [2], Kyu-Chan Shim [1], Cheryl Adeva [1], Hyun-Sook Lee [1, 3], and Sang-Nag Ahn [1,*]

[1] Department of Agronomy, Chungnam National University, Daejeon 34134, Korea; luongngocha.biotech@gmail.com (N.H.L.); zktnrl@naver.com (K.-C.S.); ccadeva_758@yahoo.com (C.A.)

[2] Agricultural Genetics Institute, North Tu Liem District, Hanoi City, Vietnam; lehunglinhbio@gmail.com (L.-H.L.)

[3] Present address: Crop Breeding Division, National Institute of Crop Science, Wanju-Gun 55365, Korea; Leehs0107@gmail.com

* Correspondence: ahnsn@cnu.ac.kr; Tel.: +82-42-821-5728

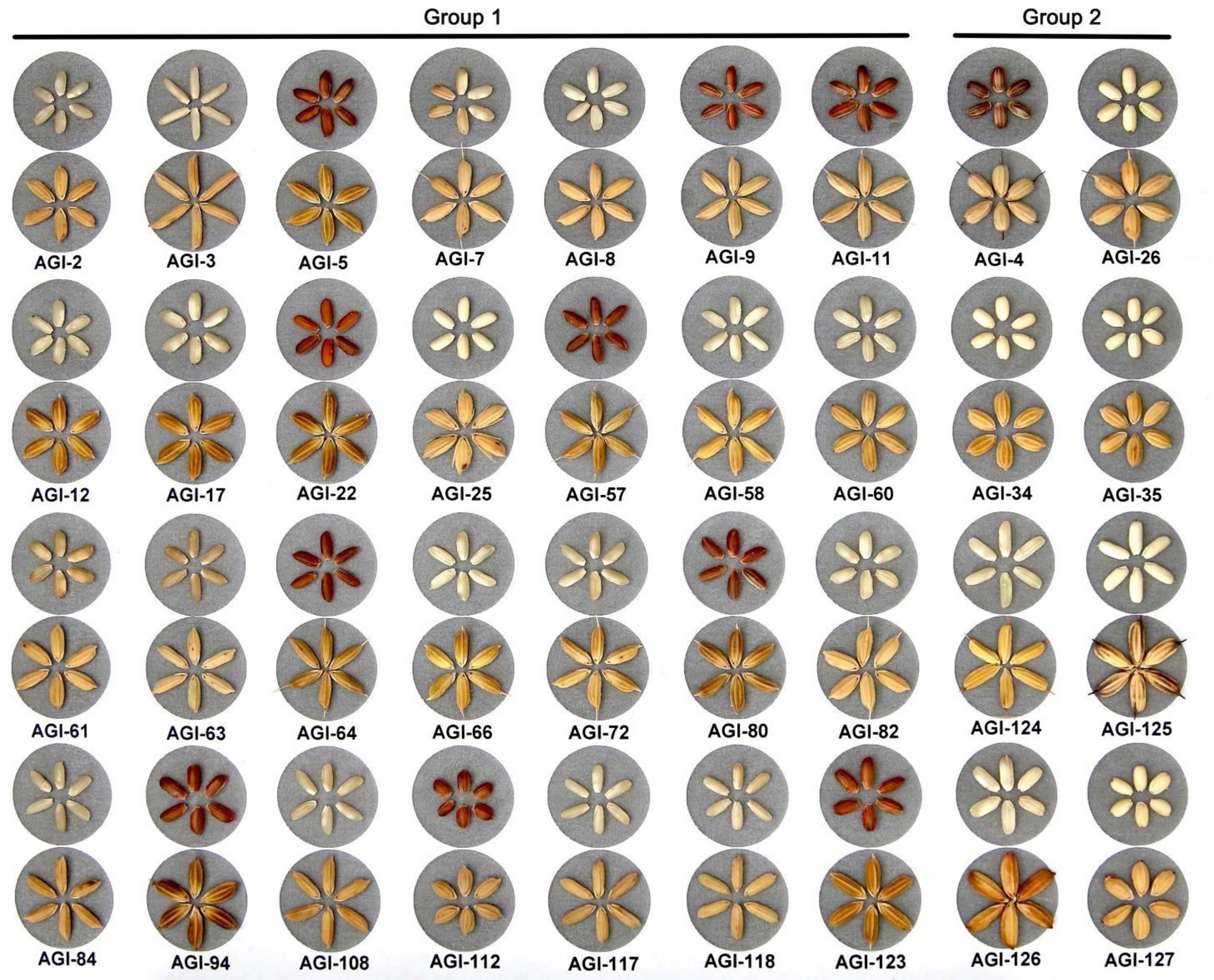

Figure S1. Photograph showing various appearances of seeds and grain pericarp in rice landraces representative for Group 1 and Group 2. The gray circle with diameter 2.2 cm used as a scale bar

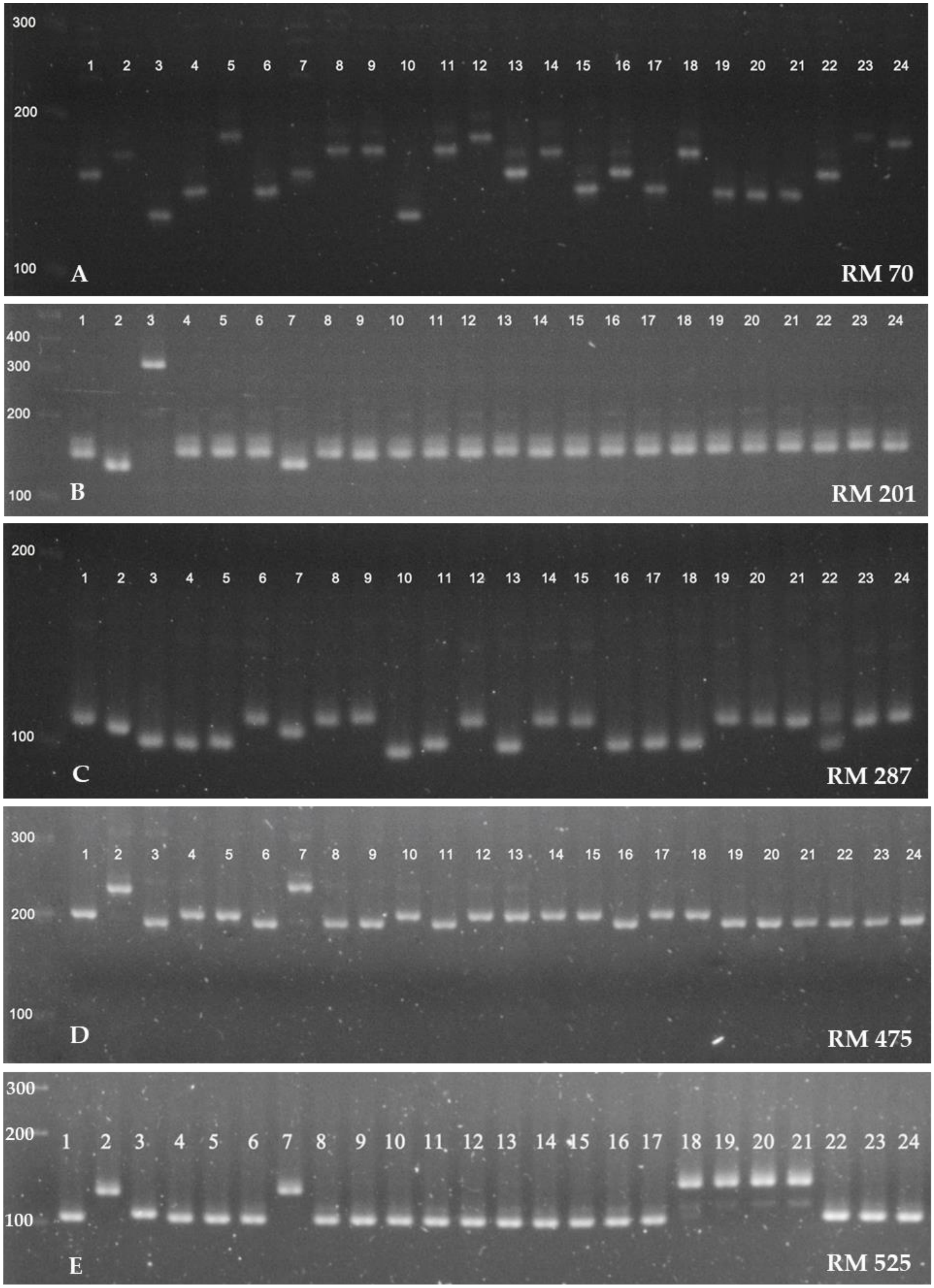

Figure S2. Gel pictures for banding of the 24 representative rice accessions with the markers: RM70 (A), RM201 (B), RM287 (C), RM475 (D), and RM525 (E). #1 - Milyang 23; #2 - Nipponbare; #3 - *O. rufipogon*; from #4 to #24: AGI-1 to AGI-24

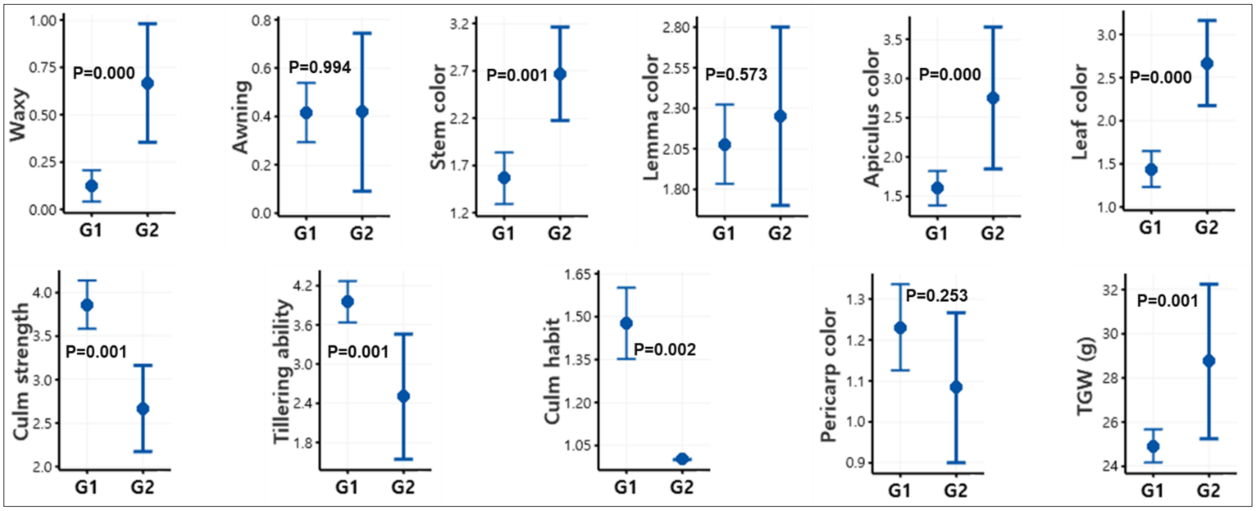

Figure S3. Comparison of the means for 11 agro-morphological traits between two subgroups by t-tests

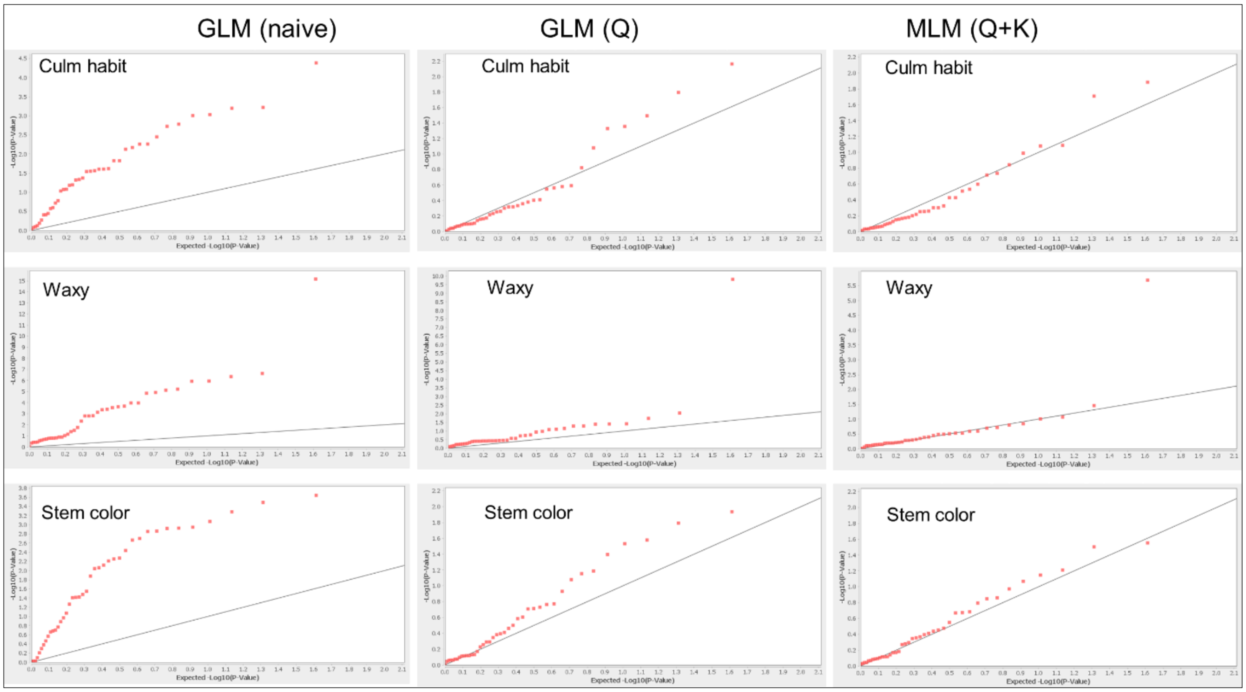

Figure S4. Quantile–quantile (Q–Q) plot of the marker-trait association by naive GLM, GLM with Q and MLM with Q + K for culm habit, waxy, and stem color
